# tRF‐59:76‐Arg‐ACG‐1‐M2 is upregulated during colorectal carcinogenesis and promotes cell proliferation, migration, and invasion

**DOI:** 10.1002/ctm2.70378

**Published:** 2025-06-30

**Authors:** Xiangrong Gao, Hao Bai, Yiming Wang, Zhaohui Zhang, Tingting Lin, Hao Fu, Jianhao Xu, Xinglin Fei, Jinhua Yang, Jinghao Sheng, Xiaojiang Ying, Lihua Zhang, Mengling Tang, Jianbing Wang, Kun Chen, Mingjuan Jin

**Affiliations:** ^1^ Department of Public Health Second Affiliated Hospital, Zhejiang University School of Medicine Hangzhou China; ^2^ Institute of Environmental Medicine, Zhejiang University School of Public Health Hangzhou China; ^3^ Jiashan Institute of Cancer Prevention and Treatment Jiaxing China; ^4^ Department of Anorectal Surgery Shaoxing People's Hospital Shaoxing China; ^5^ Department of Public Health Fourth Affiliated Hospital, Zhejiang University School of Medicine Hangzhou China; ^6^ Department of Public Health National Clinical Research Center for Child Health of Children's Hospital Zhejiang University School of Medicine Hangzhou China

1

Dear Editor

Colorectal cancer (CRC) ranks third in incidence and second in mortality worldwide as of 2022.^1^ Although CRC is driven by the accumulation of diverse molecular alterations,^2^, ^3^ its underlying mechanisms have not been fully understood. Transfer RNA‐derived small RNAs (tsRNAs) participate in diverse physiological and pathological processes by modulating gene expression at both the transcription and post‐transcription levels.^4^, ^5^ Evidence indicates that dysregulation of tsRNAs is widespread across various human diseases and plays a critical role in cancer development.^6^, ^7^, ^8^ Here, we aimed to profile dynamic changes across all stages of colorectal carcinogenesis in human and to elucidate the biological functions of tRF‐59:76‐Arg‐ACG‐1‐M2 through experiments.

A multi‐stage study was conducted, encompassing a discovery stage, two validation stages (I and II), in vitro and in vivo experiments, and bioinformatics analyses (Figure S1). Human plasma samples were obtained from the Jiashan cohort, a population‐based cohort of CRC in southeast China.^9^ We included healthy controls (HC), non‐advanced adenoma (NAA), advanced adenoma (AA), and CRC patients between June 2016 and September 2021, matched by age and sex. Follow‐up was continued until death or December 31, 2024.

In the discovery stage, small RNA sequencing was performed to profile plasma tsRNA expression and identify differentially expressed tsRNAs (fold change ≥1.50, *p* < .05) (**Table** **S1**). The proportion distribution of subtypes and the number of subtypes mapped to tRNA isodecoders across HC, adenoma and cancer groups are presented in **Figure** **S2**. tRF‐5c was the most abundant subtype in all samples (Figure 1A). We observed significant differences in the expression of 24 tsRNAs between CRC and adenoma, and of 25 tsRNAs between CRC and HC (Figure 1B). Among them, 10 tsRNAs exhibiting higher levels in the CRC group overlapped (Figure 1C,D; **Table** **S2**). These tsRNAs were selected as candidates for further validation. Nevertheless, primers were only available for 8 of them (**Table** **S3**).

**FIGURE 1:**
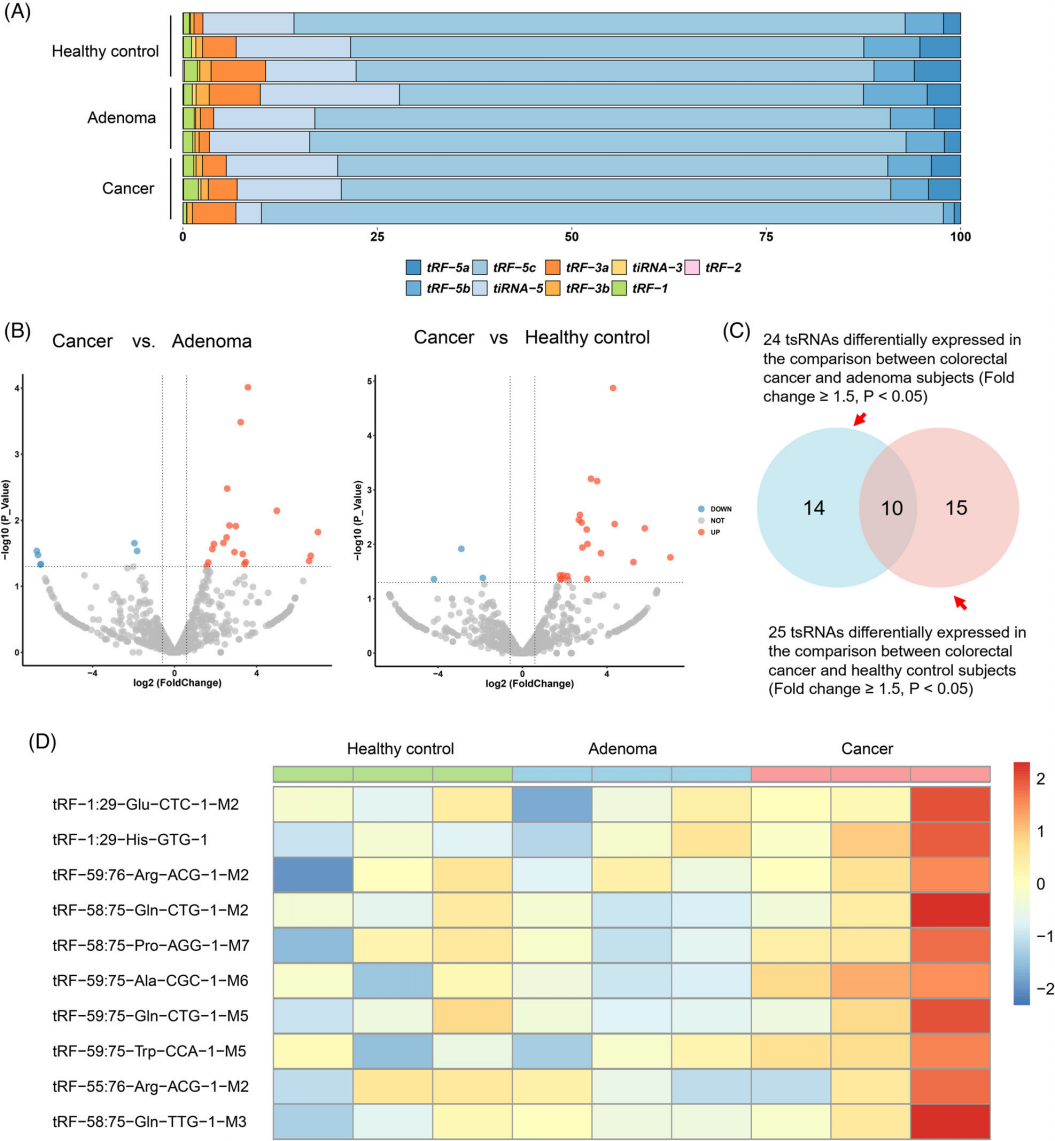
Identification of differentially expressed plasma tsRNAs in colorectal cancer patients. (A) Percentage bar showing the proportion of each sub‐type. (B) Volcano plots presenting differentially expressed tsRNAs in the comparisons of cancer versus adenoma and cancer versus healthy control. (C) Venn diagram giving the intersection of differentially expressed tsRNAs in the previous comparisons (candidate tsRNAs). (D) Heat map illustrating the relative expression levels of candidate tsRNAs in three healthy controls, three adenoma patients, and three cancer patients.

In addition to the examination of candidate tsRNAs expression across different stages of colorectal carcinogenesis, a two‐stage qRT‐PCR was conducted. The baseline characteristics did not differ significantly across the four groups in both validation stage I and II (*p* > .05) (Table 1). In validation stage I, the expression levels of the 8 candidate tsRNAs were quantified among 70 HC, 70 NAA, 70 AA and 70 CRC subjects. tRF‐59:76‐Arg‐ACG‐1‐M2 and tRF‐59:76‐Pro‐AGG‐1‐M8 showed significant differences in expression levels across the four groups (*p *< .05), while the remaining six did not (Figure 2A). The expression levels of these two tsRNAs were further measured in the validation stage II using a larger independent sub‐cohort, consisting of 87 HC, 87 NAA, 87 AA and 87 CRC subjects. As illustrated in Figure 2B, the significantly differential expression was confirmed only for tRF‐59:76‐Arg‐ACG‐1‐M2 (*p* < .05). Furthermore, its expression level progressively increased from HC to NAA, to AA, and then followed by a very slight decrease in CRC (still higher than NAA, *p*_for trend_ < .05). To assess the prognostic value of tRF‐59:76‐Arg‐ACG‐1‐M2, Kaplan–Meier survival analysis was performed among 157 CRC patients pooled from both validation stages. Although patients with higher expression levels of tRF‐59:76‐Arg‐ACG‐1‐M2 tended to have worse overall survival, the difference was not statistically significant (log‐rank *p* = .102) (Figure 2C).

**TABLE 1:** Baseline characteristics of subjects in validation stages

Index	Validation stage I					Validation stage Ⅱ				
	HC (*n* = 70)	NAA (*n* = 70)	AA (*n* = 70)	CRC (*n* = 70)	*p‐*value	HC (*n* = 87)	NAA (*n* = 87)	AA (*n* = 87)	CRC (*n* = 87)	*p‐*value
Age, year, mean (SD)	62.24 ± 6.71	62.41 ± 6.77	62.30 ± 6.85	62.54 ± 7.04	.994	62.32 ± 7.87	62.61 ± 7.85	62.10 ± 7.88	63.34 ± 8.11	.749
Sex, *n* (%)					1.000					1.000
Male	35 (50.00)	35 (50.00)	35 (50.00)	35 (50.00)		54 (62.07)	54 (62.07)	54 (62.07)	54 (62.07)	
Female	35 (50.00)	35 (50.00)	35 (50.00)	35 (50.00)		33 (37.93)	33 (37.93)	33 (37.93)	33 (37.93)	
Body mass index, *n* (%)					.751					.457
<18.5 kg/m^2^	4 (5.71)	3 (4.29)	3 (4.29)	3 (4.29)		6 (6.90)	3 (3.45)	3 (3.45)	2 (2.30)	
18.5 to <24 kg/m^2^	43 (61.43)	38 (54.29)	46 (65.71)	39 (55.71)		51 (58.62)	52 (59.77)	42 (48.28)	43 (49.43)	
24 to <28 kg/m^2^	22 (31.43)	25 (35.71)	17 (24.29)	22 (31.43)		22 (25.29)	29 (33.33)	33 (37.93)	33 (37.93)	
≥28 kg/m^2^	1 (1.43)	3 (4.29)	4 (5.71)	4 (5.71)		8 (9.20)	3 (3.45)	8 (9.20)	8 (9.20)	
Middle school and above education level, *n* (%)	27 (38.57)	26 (37.14)	19 (27.14)	21 (30.00)	.162	35 (40.23)	35 (40.23)	24 (27.59)	33 (37.93)	.377
Current married, *n* (%)	67 (95.71)	65 (92.86)	61 (87.14)	65 (92.86)	.290	78 (89.66)	86 (98.85)	79 (90.80)	78 (89.66)	.066
Current smokers, *n* (%)	20 (28.57)	24 (34.29)	26 (37.14)	27 (38.57)	.612	27 (31.03)	33 (37.93)	40 (45.98)	29 (33.33)	.182
Current drinkers, *n* (%)	17 (24.29)	16 (22.86)	21 (30.00)	17 (24.29)	.774	26 (29.89)	22 (25.29)	31 (35.63)	26 (29.89)	.527
Regular physical activity, *n* (%)	7 (10.00)	14 (20.00)	11 (15.71)	4 (5.71)	.106	18 (20.69)	12 (13.79)	15 (17.24)	26 (29.89)	.053
Family history of CRC	7 (10.00)	10 (14.29)	4 (5.71)	7 (10.00)	.414	13 (14.94)	17 (19.54)	9 (10.34)	8 (9.20)	.172
Regular aspirin use	0 (0.00)	3 (4.29)	1 (1.43)	1 (1.43)	.276	8 (9.20)	3 (3.45)	3 (3.45)	6 (6.90)	.282

Abbreviations: AA, advanced adenoma; CRC, colorectal cancer; HC, healthy control; NAA, non‐advanced adenoma.

**FIGURE 2:**
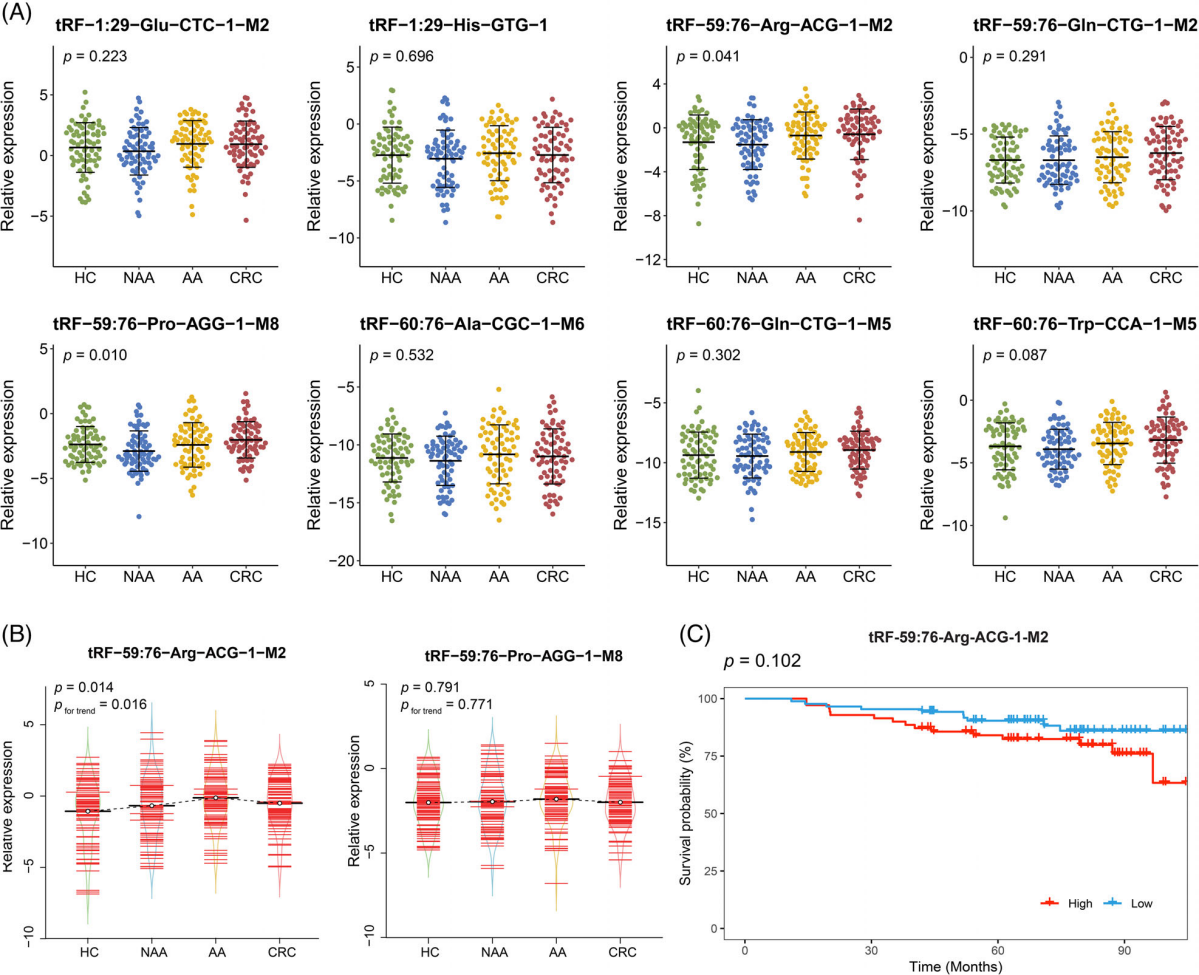
Validation of differences in candidate tsRNAs expression levels among HC, NAA, AA, and CRC groups. (A) Scatter plots showing relative expression levels of 8 candidate tsRNAs by qRT‐PCR among 70 HC, 70 NAA, 70 AA and 70 CRC subjects in validation stage I. (B) Bean plots representing relative expression levels of tRF‐59:76‐Arg‐ACG‐1‐M2 and tRF‐59:76‐Pro‐AGG‐1‐M8 by qRT‐PCR among 87 HC, 87 NAA, 87 AA, and 87 CRC subjects in validation stage II. The distribution of individual subjects is presented as small red horizontal lines, and the mean value of each group is shown as a solid black horizontal line. (C) Kaplan‐Meier survival curves among all CRC patients stratified by tRF‐59:76‐Arg‐ACG‐1‐M2 expression levels. AA, advanced adenoma; CRC, colorectal cancer; HC, healthy control; NAA, non‐advanced adenoma.

Then, we further explored the functional role of tRF‐59:76‐Arg‐ACG‐1‐M2 through cell experiments. The transfection efficiency of its mimic and inhibitor was confirmed in RKO and HCT116 cells (Figure 3A). The CCK8 assay revealed that the mimic significantly enhanced cell proliferation, whereas the inhibitor suppressed it (Figure 3B). Wound healing and transwell assays demonstrated that the mimic promoted cell migration, while the inhibitor reduced it (Figure 3C,D). Additionally, the transwell assay with Matrigel showed that the overexpression of tRF‐59:76‐Arg‐ACG‐1‐M2 increased CRC cell invasion, whereas its downregulation inhibited invasion (Figure 3D). Taken together, these results demonstrated that tRF‐59:76‐Arg‐ACG‐1‐M2 promotes colorectal carcinogenesis. To further validate its in vivo function, we performed animal experiments using a xenograft model. HCT116 cells were injected subcutaneously into BALB/c nude mice, and after 5 days, mice were randomized to receive intratumoral injections of tRF‐59:76‐Arg‐ACG‐1‐M2 antagomir to inhibit its expression or phosphate‐buffered saline (PBS) as control (Figure 3E). In the antagomir‐treated group compared with controls, significantly slower subcutaneous tumour growth and notably reduced tumour weight after 2 weeks were observed (Figure 3F–H), and a significant decrease in the proportion of Ki‐67‐positive cells was found by subsequent immunohistochemistry (IHC) staining on the subcutaneous tumours (**Figure** **S3**). These findings demonstrate that inhibiting tRF‐59:76‐Arg‐ACG‐1‐M2 suppresses CRC tumour growth in vivo.

**FIGURE 3:**
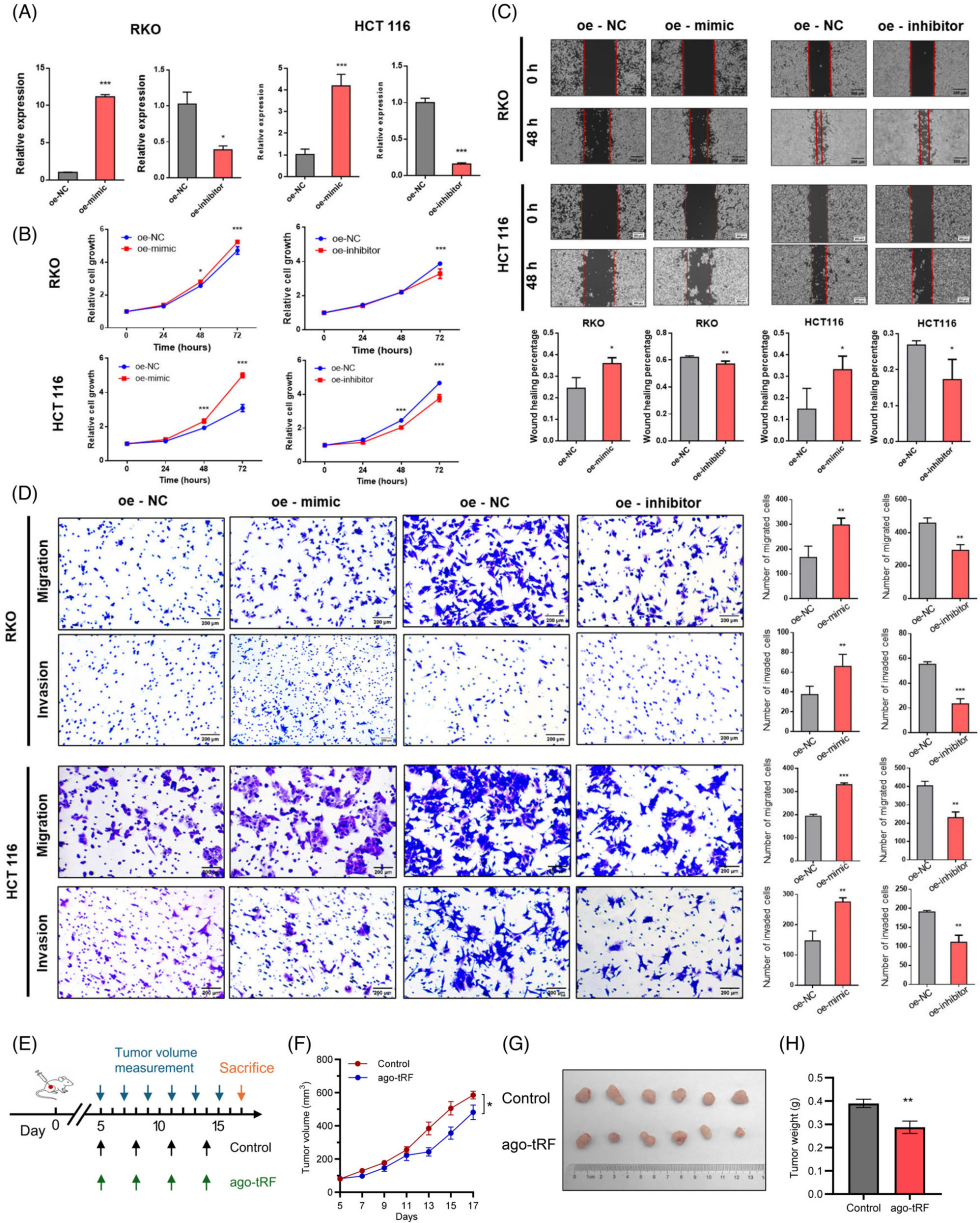
Biological functions of tRF‐59:76‐Arg‐ACG‐1‐M2 in colorectal cancer in vitro and in vivo. (A) Transfection efficiency of its mimic or inhibitor in RKO and HCT116 cells. (B) Line chart showing cell proliferation over time measured by CCK8 assay in RKO and HCT116 cells. (C) Representative images and histogram statistics from wound healing assay. (D) Representative images and histogram statistics from transwell migration and invasion assays; (E) Schematic workflow illustrating the establishment of subcutaneous xenograft models overexpressing tRF‐59:76‐Arg‐ACG‐1‐M2. (F) Tumour growth curves depicting the progression of subcutaneous tumour volume over time. (G) Photographs of excised tumours from nude mice at endpoint. (H) Bar chart summarizing tumour weights.

Secondary structure modelling classified tRF‐59:76‐Arg‐ACG‐1‐M2 as a tRF‐3a type generated by cleavage of tRNA‐Arg‐ACG‐1 (Figure 4A). A total of 539 overlapping target genes were identified through the prediction by miRDB and miRanda (Figure 4B). Gene Ontology (GO) analysis highlighted roles in the regulation of transmembrane transporter activity, asymmetric synapses, and channel regulator activity (Figure 4C). Kyoto Encyclopedia of Genes and Genomes (KEGG) pathway enrichment analysis implicated MAPK, PI3K‐Akt, and calcium signalling pathways (Figure 4D). These findings suggest that tRF‐59:76‐Arg‐ACG‐1‐M2 may play a crucial role in CRC development and progression.

**FIGURE 4:**
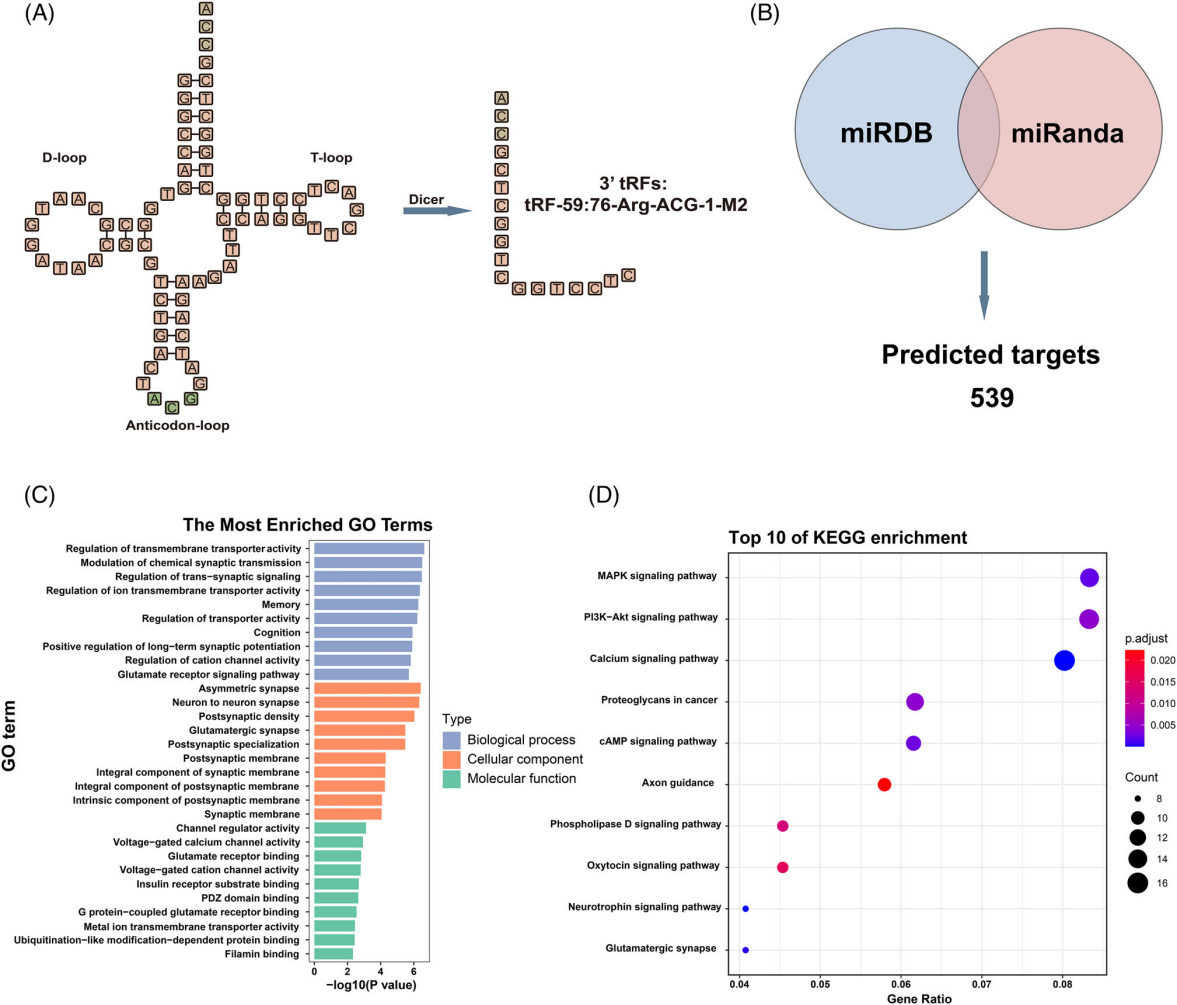
Biogenesis and enrichment analysis of predicted target genes of tRF‐59:76‐Arg‐ACG‐1‐M2. (A) Secondary structure prediction. (B) Venn diagram displaying the intersection of predicted target genes in miRDB and miRanda. (C) The predicted targets of GO analysis. (D) The predicted targets of KEGG analysis.

In conclusion, this study identified that tRF‐59:76‐Arg‐ACG‐1‐M2 has an increasing pattern across all stages of colorectal carcinogenesis. Functional assays revealed its role in promoting cell proliferation, migration, and invasion, and bioinformatics tools also validated its involvement in CRC development and progression. These results suggest that tRF‐59:76‐Arg‐ACG‐1‐M2 holds great potential as a biomarker for CRC diagnosis and prognosis and deserves further investigation.

## AUTHOR CONTRIBUTIONS

Mingjuan Jin conceived and designed the study. Xiangrong Gao and Hao Bai performed the experiments with human samples, analyzed the data, and drafted the manuscript. Zhaohui Zhang performed the cell experiments. Tingting Lin and Yiming Wang conducted the animal experiments. Hao Fu, Jianhao Xu, Xinglin Fei, Jinhua Yang, Xiaojiang Ying, and Lihua Zhang recruited the study participants and collected the samples. Xiangrong Gao and Hao Bai drafted the manuscript under the supervision of Mingjuan Jin and Jinghao Sheng. Kun Chen, Mengling Tang, and Jianbing Wang revised the manuscript critically. All authors read and approved the final manuscript.

## CONFLICT OF INTEREST STATEMENT

The authors declare no conflict of interest.

## ETHICS STATEMENT

This study was approved by the ethics committee of Zhejiang University School of Medicine (Hangzhou, China). Written informed consent was obtained from all participants, and data were analyzed anonymously. All animal experimental procedures and protocols were approved by the Medical Experimental Animal Care Commission of Zhejiang University.

## Supporting information



Supporting Information

## Data Availability

The datasets used during the current study are available from the corresponding author upon reasonable request.
