# Supplementary material for: tRF‐59:76‐Arg‐ACG‐1‐M2 is upregulated during colorectal carcinogenesis and promotes cell proliferation, migration, and invasion
Source: Clin Transl Med. 2025 Jun 30;15(7):e70378. doi: 10.1002/ctm2.70378 (PMC12209506; doi:10.1002/ctm2.70378)
Supplement: Supplementary file 1 — Supporting Information [file CTM2-15-e70378-s001.docx]

**Supplementary materials**

**Materials and methods**

**Study population**

All cases of colorectal cancer (CRC), advanced adenoma (AA) and non-advanced adenoma (NAA) cases were histologically confirmed. For adenomas, AA was defined as those with a diameter ≥ 10 mm, high-grade dysplasia, or villous/tubulovillous histology, while the remaining adenomas were classified as NAA according to guidelines^1^. CRC plasma samples in discovery stage were collected from Shaoxing People’s Hospital, China^2^. All other plasma samples of study subjects were obtained from Jiashan cohort, China. This research received approval from the Medical Ethics Committee of Zhejiang University School of Medicine, and written informed consent was obtained from all participants

**Sample collection**

A 5 mL sample of whole blood was collected from each study participant using a disposable EDTA anticoagulant blood collection tube, stored at 4°C and centrifuged within 2 hours at 12,000 rpm for 10 minutes in a high-speed refrigerated centrifuge to separate the plasma and blood cells. The upper pale-yellow plasma layer was then carefully collected, aliquoted into sterile RNase-free storage tubes, and stored at -80°C. All participants were pathologically confirmed to have no familial adenomatous polyposis (FAP), no previous history of CRC, and no preoperative anticancer treatment.

**Total RNA isolation, library preparation and sequencing**

Total RNA was extracted for small RNA-seq using TRIzol LS reagent (Invitrogen, Carlsbad, CA, USA). The RNA samples were pretreated with 3’-aminoacyl diacylation, 5’-OH phosphorylation, and m1A and m3C demethylation to remove RNA modifications that interfere with sequencing. PCR-amplified fragments were extracted and purified from the PAGE gels. Libraries were qualified and quantified using an Agilent BioAnalyzer 2100. Sequencing was then conducted on an Illumina NextSeq 500 platform using 50bp single-read configuration.

Sequencing quality was examined with FastQC and adapter trimming, and low-quality reads were filtered using Cutadapt^3^. Reads were aligned to the tRNA sequences with Bowtie software^4^, allowing for one mismatch.

**Small RNA isolation and qRT-PCR**

Small RNA was isolated from plasma using the miRcute serum/plasma miRNA isolation kit (DP503, Tiangen Biotech Co., Ltd., Beijing, China) and from cells was isolated using the miRcute miRNA isolation kit (DP501, Tiangen Biotech Co., Ltd., Beijing, China) following the manufacturer’s instruction. The expression levels of specific tsRNAs were normalized to Cel-miR-39 or U6, respectively. Stem-loop qPCR was conducted to detect target tsRNAs^5^. cDNA synthesis was performed with Reverse Transcriptase M-MLV (Takara, Kyoto, Japan). Subsequently, qRT-PCR reactions were carried out using SYBR Premix Ex Taq™ (Takara, Kyoto, Japan) and run with a Roche Light Cycler 480 II system (Roche, Germany).

**Cell culture and transfection**

Human CRC cell line RKO and HCT116 were cultured in DMEM supplemented with 10% fetal bovine serum (FBS) and 1% antibiotics and in a thermostatic incubator at 37°C with 5% CO_2_. The single-stranded mimics and inhibitors of tRF-59:76-Arg-ACG-1-M2 were constructed by Shanghai GenePharma Co., Ltd. All of these at a final concentration of 50nM were transfected using Lipofectamine^TM^ 2000 Transfection Reagent (Invitrogen, USA) following the manufacturer’s protocol. tRF-59:76-Arg-ACG-1-M2 levels were measured 24 hours post-transfection.

**Cell proliferation assay**

Cell proliferation was assessed by using a Cell Counting Kit 8 (CCK8; Beyotime Biotechnology, Shanghai, China) following the manufacturer’s instructions. Transfected CRC cells were seeded in 96-well plates at an initial density of 2000 cells/well. The tests were performed at 0, 24, 48, and 72 hours. Ten microliters of CCK8 solution was added to each well, and the plates were incubated at 37℃ for 3 hours. Then, the absorbance was measured at 450 nm using a microplate reader (Bio-Rad, Richmond, CA, USA).

**Wound healing assay**

Transfected RKO and HCT116 were seeded in 6-well plates. After reaching 100% confluence, a scratch was made in the center of each well using a sterile 200 μL pipette tip. Cells were rinsed twice with PBS buffer, followed by the addition of serum-free medium. Images of the wound margins were captured at 0 and 48 hours using an inverted microscope (Olympus CKX53, Japan). The blank area between the wound margins was measured.

**Transwell assay**

Migration and invasion assays were conducted in 8-µm transwell chambers (BIOFIL, Guangzhou, China). For migration assay, 1.5×10^5^ (for mimics) or 2.0×10^5^ (for inhibitors) cells in serum-free medium were seeded into the upper chamber. The cells were cultured in a 24-well plate containing 600 μL of 10% serum medium. After 48 hours, cells were fixed in 4 % paraformaldehyde and stained with 0.1% crystal violet. The non-migrating cells in the upper chamber were removed using a cotton swab, and the migrated cells on the lower surface were photographed under an inverted microscope and counted. For the invasion assay, the upper chamber was precoated with 60μL Matrigel (Corning, USA), and all other processes were the same.

**Animal experiments**

Tumorigenesis assay was performed using 5-week-old BALB/c nude mice. HCT116 cells (1.0×10^6^ in 100 μL phosphate-buffered saline [PBS]) were injected subcutaneously through the right axilla. Five days after tumor cell inoculation, mice were randomly divided into two groups (n = 6 per group): tRF-59:76-Arg-ACG-1-M2 antagomir and PBS control. Each mouse received 2.5 nmol of RNA oligonucleotides (GenePharma Co., Ltd, Shanghai, China) via multi-point intratumoral injections, administered four times at 3-day intervals. The tumors’ length (a) and width (b) were measured every 2 days using digital caliper, and the volume was calculated as *V* = ab^2^/2. After 2 weeks, mice were sacrificed and tumors were excised for weight measurements and subsequent analyses. All animals were maintained under specific pathogen-free (SPF) conditions at 18–23 °C with 40–60% humidity and a standard 12-hour light/dark cycle at the Laboratory Animal Centre of Zhejiang University. Mice were provided with water and a standard laboratory diet ad libitum. All procedures complied with the guide for the care and use of laboratory animals and were approved by the Medical Experimental Animal Care Commission of Zhejiang University.

**Functional enrichment analysis**

Gene Ontology (GO, http://www.geneontology.org) and Kyoto Encyclopedia of Genes and Genomes database (KEGG, www.genome.jp/kegg) were used to show cellular and molecular function and significant pathways of the target genes. GO and KEGG terms with *p* < .05, a minimum count of 3 and an enrichment factor of >1.5 were considered significant. False discovery rates (FDR) were calculated to correct the *p* values based on an accumulative hypergeometric distribution.

**Statistical analysis**

Continuous variables were reported as mean and standard deviation (SD), and categorical data as numbers and percentages. Differentially expressed tsRNAs were analyzed using the edgeR package in R^6^. |Log_2_ (Fold change [FC]) | ≥ log_2_1.5 and *p* < .05 were used to identify significantly differentially expressed tsRNAs. Differences among healthy controls (HC), NAA, AA and CRC groups were analyzed using the chi-square test for categorical variables and one-way ANOVA for continuous variables. Liner modelling was used to examine changes in tsRNA expression levels across the different stages of colorectal carcinogenesis. In the survival analysis, the optimal cutoff value for tRF-59:76-Arg-ACG-1-M2 expression was determined using the surv_cutpoint() function from the *survminer* package, with a minimum group proportion of 0.4. Based on this cutoff, patients were stratified into high-expression and low-expression groups. Survival differences between groups were evaluated using Kaplan–Meier analysis and compared with the log-rank test. Statistical analysis was performed with GraphPad Prism software (version 10.0) and R software (version 4.4.1).

**Reference**

1. Rex DK, Boland CR, Dominitz JA, et al. Colorectal cancer screening: recommendations for physicians and patients from the U.S. multi-society task force on colorectal cancer. *The American Journal of Gastroenterology*. 2017;112(7):1016-1030.

2. Ye D, Huang Q, Li Q, et al. Comparative Evaluation of Preliminary Screening Methods for Colorectal Cancer in a Mass Program. *Digestive Diseases and Sciences*. 2017;62(9):2532-2541.

3. Martin M. CUTADAPT removes adapter sequences from high-throughput sequencing reads. *EMBnetjournal*. 2011;17

4. Langmead B, Trapnell C, Pop M, Salzberg SL. Ultrafast and memory-efficient alignment of short DNA sequences to the human genome. *Genome Biology*. 2009;10(3):R25.

5. Tosar JP, Gámbaro F, Darré L, Pantano S, Westhof E, Cayota A. Dimerization confers increased stability to nucleases in 5' halves from glycine and glutamic acid tRNAs. *Nucleic acids research*. Sep 28 2018;46(17):9081-9093. doi:10.1093/nar/gky495

6. Robinson MD, McCarthy DJ, Smyth GK. edgeR: a Bioconductor package for differential expression analysis of digital gene expression data. *Bioinformatics*. Jan 1 2010;26(1):139-140. doi:10.1093/bioinformatics/btp616

**Supplementary Figures**

**
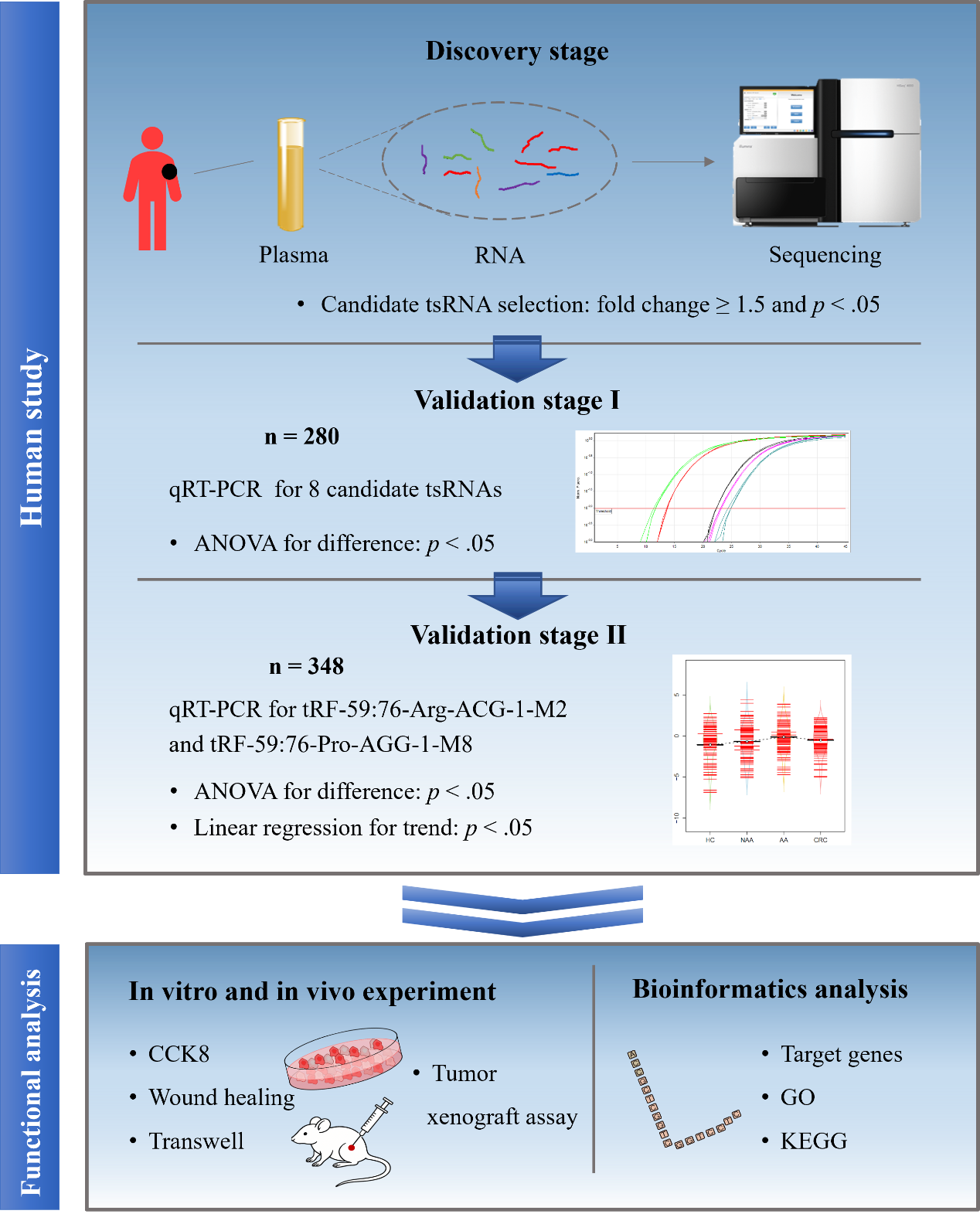
**

**Figure S1.** Flowchart of the study.


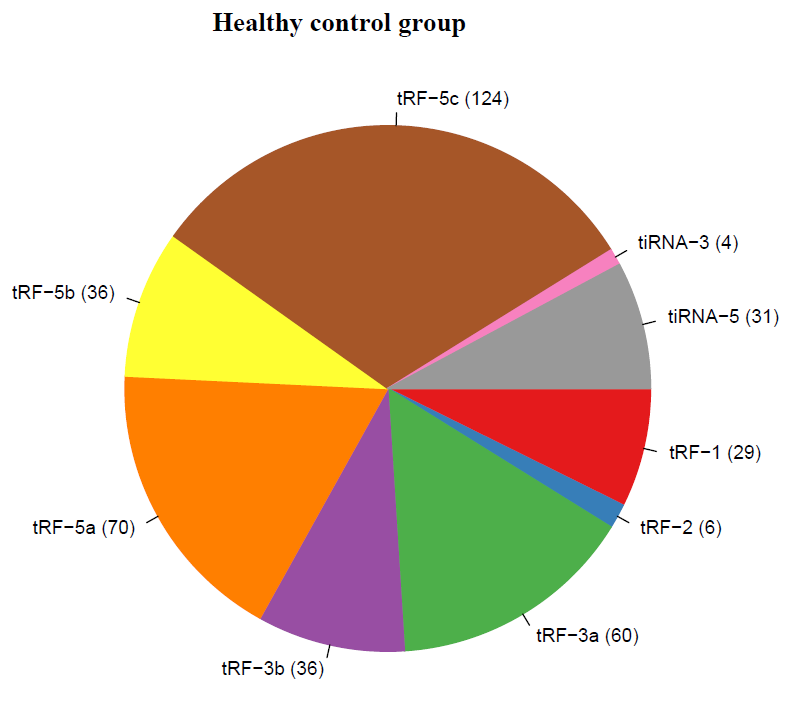

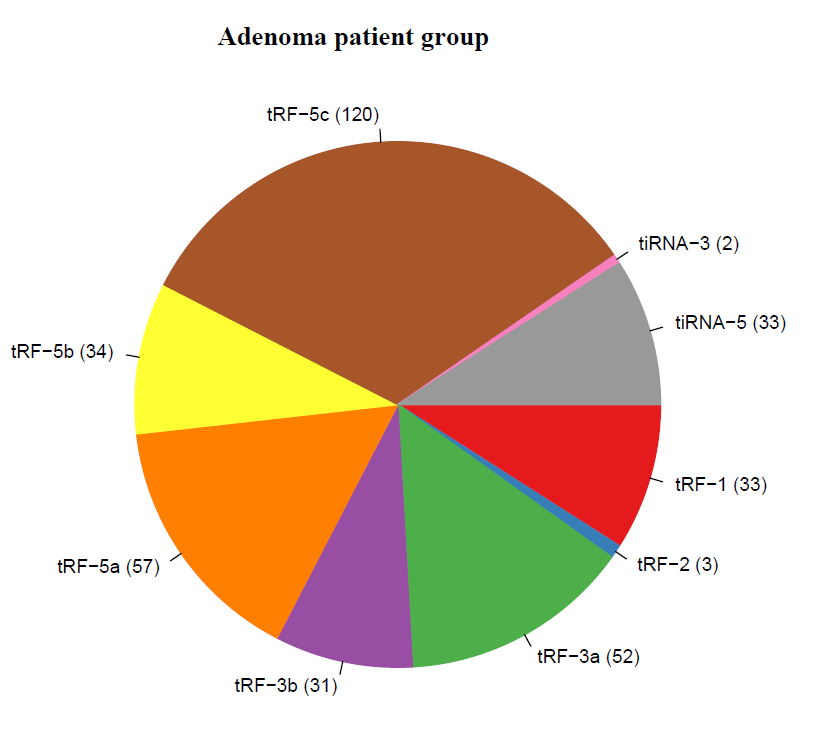

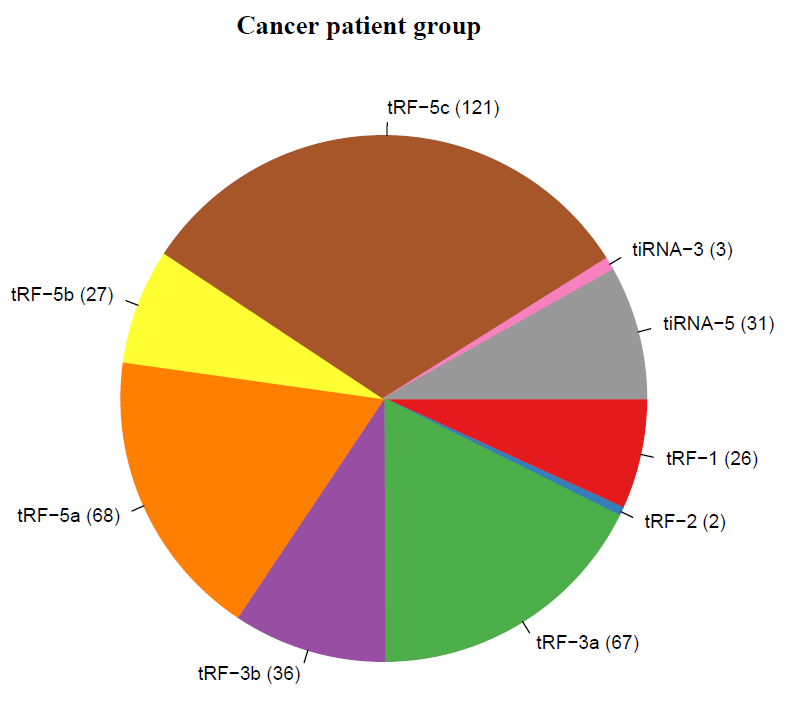


**B**

**A**


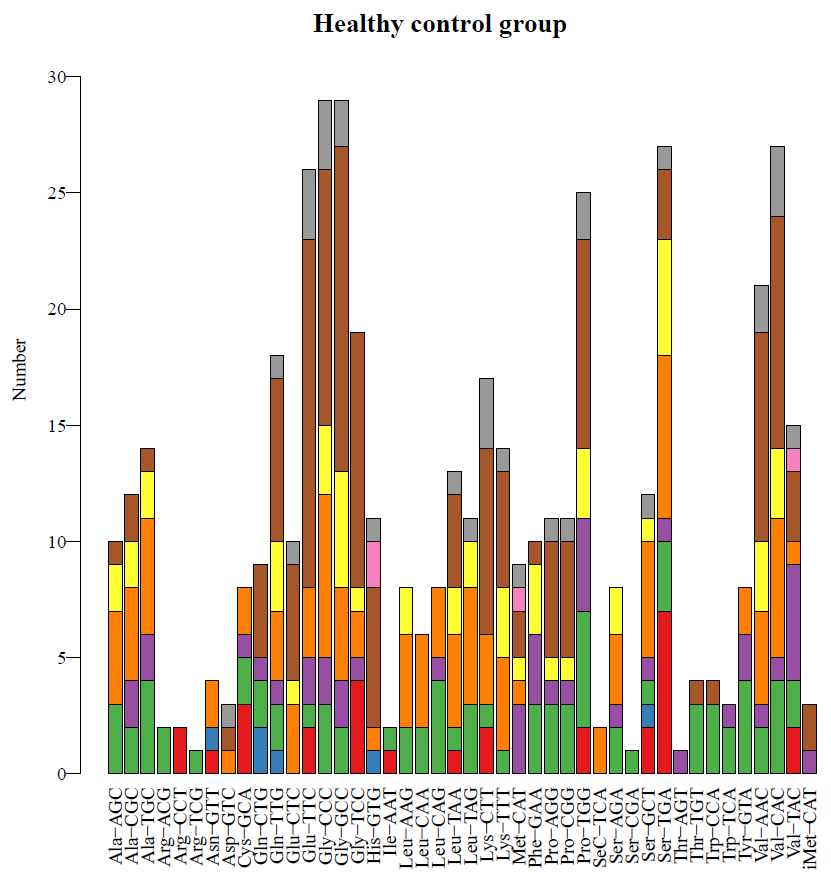

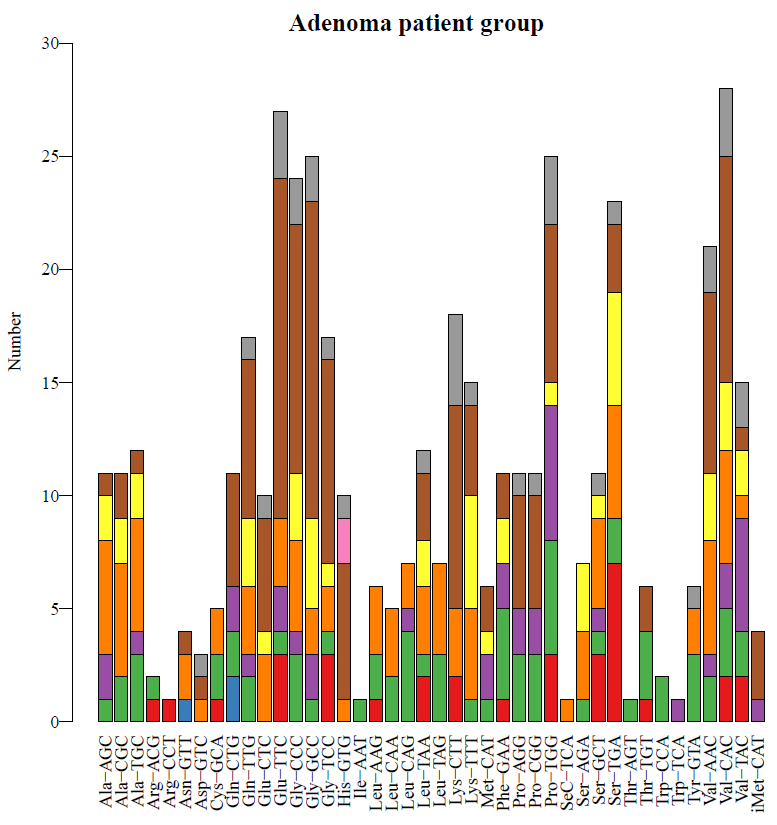

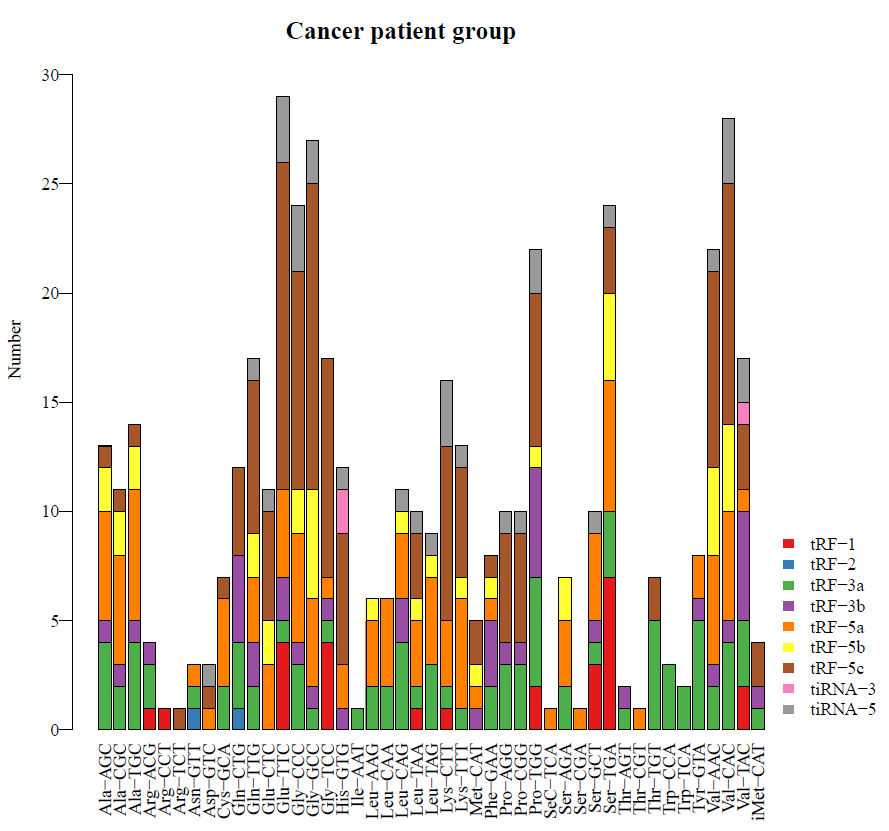


**Figure S2.** The profiles of plasma tsRNA expression across healthy control, adenoma patient, and cancer patient groups. (A) Proportion distribution of tsRNA subtypes among healthy control, adenoma patient, and cancer patient groups. (B) The number of tsRNA subtypes mapped to tRNA isodecoders across healthy control, adenoma patient, and cancer patient groups. The x-axis represents tRNA isodecoders, and the y-axis denotes the total counts of tsRNA subtypes mapped to each isodecoder, visualized as stacked bars.

**
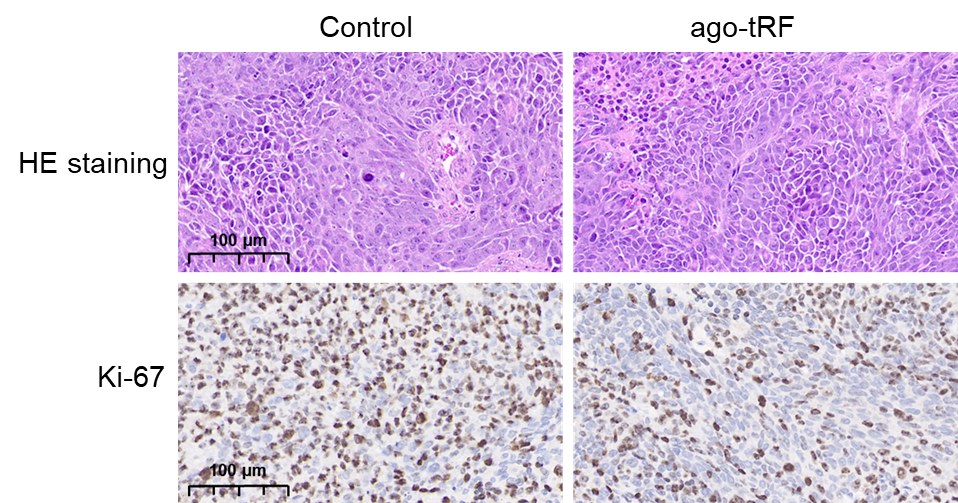
**

**Figure S3.** Haematoxylin–eosin (HE) staining (up) of tumors confirmed the successful construction of xenograft models. Immunohistochemistry (IHC) (down) of tumors indicated a lower proportion of ki-67-positive cells in ago-tRF group.

**Supplementary Tables**

**Table S1.** Basic characteristics of subjects in discovery stage

| **Group** | **No.** | **Age** | **Gender** | **Lesion set** | **Smoke** | **Alcohol** |
| --- | --- | --- | --- | --- | --- | --- |
| Cancer | 1 | 70 | Female | Colon | Never | Never |
|  | 2 | 64 | Male | Rectum | Never | Never |
|  | 3 | 75 | Male | Colon | Never | Never |
| Adenoma | 4 | 70 | Female | Colon | Never | Never |
|  | 5 | 64 | Male | Rectum | Never | Never |
|  | 6 | 75 | Male | Colon | Never | Never |
| Healthy control | 7 | 70 | Female | - | Never | Never |
|  | 8 | 64 | Male | - | Never | Never |
|  | 9 | 75 | Male | - | Never | Never |

| **Table S2** Significantly differentially expressed tsRNAs in the comparisons of cancer versus adenoma and cancer versus healthy control | | | | | | | |
| --- | --- | --- | --- | --- | --- | --- | --- |
| **tsRNA ID** | **Type** | **Length** | **Cancer vs. adenoma** | |  | **Cancer vs. healthy control** | |
|  |  |  | **Log_2_ FC** | ***p*** |  | **Log_2_ FC** | ***p*** |
| tRF-1:29-Glu-CTC-1-M2 | 5c-tRF | 29 | 1.78 | 0.037 |  | 1.57 | 0.049 |
| tRF-1:29-His-GTG-1 | 5c-tRF | 29 | 1.79 | 0.044 |  | 2.57 | 0.003 |
| tRF-59:76-Arg-ACG-1-M2 | 3a-tRF | 18 | 3.04 | 0.005 |  | 2.67 | 0.012 |
| tRF-59:76-Gln-CTG-1-M2 | 3a-tRF | 18 | 4.29 | 1.34×10^-5^ |  | 3.23 | 3.28×10^-4^ |
| tRF-59:76-Pro-AGG-1-M8 | 3a-tRF | 18 | 2.7 | 0.003 |  | 1.84 | 0.027 |
| tRF-60:76-Ala-CGC-1-M6 | 3a-tRF | 17 | 2.84 | 0.011 |  | 2.38 | 0.022 |
| tRF-60:76-Gln-CTG-1-M5 | 3a-tRF | 17 | 2.67 | 0.004 |  | 1.92 | 0.023 |
| tRF-60:76-Trp-CCA-1-M5 | 3a-tRF | 17 | 2.17 | 0.046 |  | 2.54 | 0.018 |
| tRF-55:76-Arg-ACG-1-M2^†^ | 3b-tRF | 22 | 4.37 | 0.004 |  | 2.92 | 0.03 |
| tRF-58:75-Gln-TTG-1-M3^†^ | 3a-tRF | 18 | 3.25 | 0.001 |  | 3.58 | 9.71×10^-5^ |
| ^†^The primer design was failed.  Abbreviation: FC, fold change. | | | | | | | |

| **Table S3.** The primer sequences of Cel-miRNA-39, U6 and candidate tsRNAs | | | |
| --- | --- | --- | --- |
| **tsRNA ID** | **Primer direction** | **Sequence** | **Product length (bp)** |
| Cel-miRNA-39 | Forward | ATATCATCTCACCGGGTGTAAATC | 67 |
|  | Reverse | TATGGTTTTGACGACTGTGTGAT |  |
| U6 | Forward | CAGCACATATACTAAAATTGGAACG | 76 |
|  | Reverse | ACGAATTTGCGTGTCATCC |  |
| tRF-59:76-Arg-ACG-1-M2 | Forward | TGTATGCTACTCCTGGCTGGC | 69 |
|  | Reverse | TATCCTTCTTCACGACTCCTTCAC |  |
| tRF-60:76-Trp-CCA-1-M5 | Forward | GCGTACTATTCACGTCGGGG | 68 |
|  | Reverse | TATGGTTGTAGAGCAGTGGTTGAC |  |
| tRF-59:76-Gln-CTG-1-M2 | Forward | AGTCGTAGCATCTCGGTGGAA | 69 |
|  | Reverse | TATGGTTGTAGAGCAGTGGTTGAC |  |
| tRF-60:76-Gln-CTG-1-M5 | Forward | CCTACATCGTCTCGGTGGAA | 68 |
|  | Reverse | TATGGTTGTAGAGCAGTGGTTGAC |  |
| tRF-60:76-Ala-CGC-1-M6 | Forward | TGTATCAATCCCCGGCATCT | 68 |
|  | Reverse | TATCCTTCTTCACGACTCCTTCAC |  |
| tRF-59:76-Pro-AGG-1-M8 | Forward | AGCTAGATGTATCCCGGACGAG | 62 |
|  | Reverse | GTGCAGGGTCCGAGGT |  |
| tRF-1:29-His-GTG-1 | Forward | TACAGCCGTGATCGTATAGTGGTTA | 73 |
|  | Reverse | GTGCAGGGTCCGAGGT |  |
| tRF-1:29-Glu-CTC-1-M2 | Forward | TGTAATCCCTGGTGGTCTAGTGGT | 75 |
|  | Reverse | GTGCAGGGTCCGAGGT |  |
